# Seasonal variation in environmental DNA in relation to population size and environmental factors

**DOI:** 10.1038/srep46294

**Published:** 2017-04-10

**Authors:** Andrew S. Buxton, Jim J. Groombridge, Nurulhuda B. Zakaria, Richard A. Griffiths

**Affiliations:** 1 Durrell Institute of Conservation and Ecology, School of Anthropology and Conservation, University of Kent, Marlowe Building, Canterbury, Kent, CT2 7NR, UK

## Abstract

Analysing DNA that organisms release into the environment (environmental DNA, or eDNA) has enormous potential for assessing rare and cryptic species. At present the method is only reliably used to assess the presence-absence of species in natural environments, as seasonal influences on eDNA in relation to presence, abundance, life stages and seasonal behaviours are poorly understood. A naturally colonised, replicated pond system was used to show how seasonal changes in eDNA were influenced by abundance of adults and larvae of great crested newts (*Triturus cristatus*). Peaks in eDNA were observed in early June when adult breeding was coming to an end, and between mid-July and mid-August corresponding to a peak in newt larval abundance. Changes in adult body condition associated with reproduction also influenced eDNA concentrations, as did temperature (but not rainfall or UV). eDNA concentration fell rapidly as larvae metamorphosed and left the ponds. eDNA concentration may therefore reflect relative abundance in different ponds, although environmental factors can affect the concentrations observed. Nevertheless, eDNA surveys may still represent an improvement over unadjusted counts which are widely used in population assessments but have unreliable relationships with population size.

All living organisms continually expel DNA into the environment via faeces, urine, skin secretions, skin cells and gametes^1^,^2^,^3^. The emergence of techniques that are able to detect low levels of such environmental DNA (eDNA) has enormous potential to break new ground in areas such as invasive species research^4^,^5^,^6^, pathogen detection^7^, palaeoecology^8^, and forensics and law enforcement^9^. The use of eDNA to survey rare and cryptic species that are difficult to detect using traditional methods also has wide implications for biodiversity assessment and the protection of species^10^,^11^. A relationship between the amount of eDNA present and measures of abundance has been demonstrated in both natural and mesocosm systems^6^,^12^,^13^,^14^,^15^,^16^,^17^,^18^. Although some studies suggest peaks in eDNA associated with breeding^16^,^19^,^20^, the seasonal dynamics of eDNA in relation to population size are poorly understood. Consequently, eDNA is currently largely limited to surveys of presence and absence. Measures of abundance are more useful than presence-absence, but are often based on count data that are not adjusted for detection probability which can be misleading^21^. As such, producing reliable population, biomass or relative abundance estimates would be much more informative for conservation practitioners^10^. Before predicting abundance, the factors that influence eDNA concentration in relation to changes in population size and environmental factors need to be understood.

The concentration of eDNA at any point in time will depend on (1) the rate of production of eDNA by the species; and (2) how long eDNA persists in the environment^22^. eDNA release and accumulation rates depend on a number of factors including the density of individuals, their physiology, metabolism and temperature^23^. However, eDNA can be broken down by biotic and abiotic factors such as extracellular enzymes, high temperatures, UV, and chemicals^22^,^24^,^25^,^26^,^27^,^28^,^29^. In aquatic environments, eDNA can also become incorporated into sediment^17^. Persistence of eDNA in water after organisms are removed can range from less than one day^30^, to over three weeks^22^ depending on environmental conditions, whereas persistence in soil or sediment is likely to be much longer^31^. Despite this knowledge base, and the fact that eDNA concentration can vary seasonally^19^,^20^, to our knowledge no studies have identified how seasonal population dynamics impact eDNA concentration in relation to other factors that influence DNA release and degradation. Therefore, whilst eDNA surveys promises to redefine how biodiversity is monitored in the future, there is considerable uncertainty about the relationship between eDNA concentrations and seasonal changes in population size, because of the influence of other environmental factors.

In this study we examined the relationship between eDNA and the seasonal population dynamics of great crested newts (*Triturus cristatus*) using a replicated but naturally colonised system of eight ponds. Adult great crested newts migrate into ponds to breed in the spring, with most returning to land in early summer. Breeding occurs in water with females laying eggs that hatch into aquatic larvae that metamorphose and emerge in the late summer or, occasionally, overwinter^32^. All of these stages may release eDNA into the water. As a European Protected Species, great crested newt eDNA surveys are currently being used to assess the presence-absence of species, but how eDNA fluctuates over this aquatic phase is unknown^33^. To fill this knowledge gap, adult and larval abundance, adult body condition and environmental factors including temperature, rainfall and UV, were used to evaluate their influences on eDNA concentrations throughout the aquatic period.

## Results

Between 26 February 2015 and 29 October 2015, a total of 389 captures of 49 individuals were made across the eight ponds, with capture-mark-recapture models yielding an overall population size of between 53 and 60 individuals with a most likely population size of 57, although the numbers varied between ponds. Likewise, 408 larvae were captured between 28 May 2015 and 29 October 2015, with an estimated bottle trapping detectability of 0.39.

**Figure 1:**
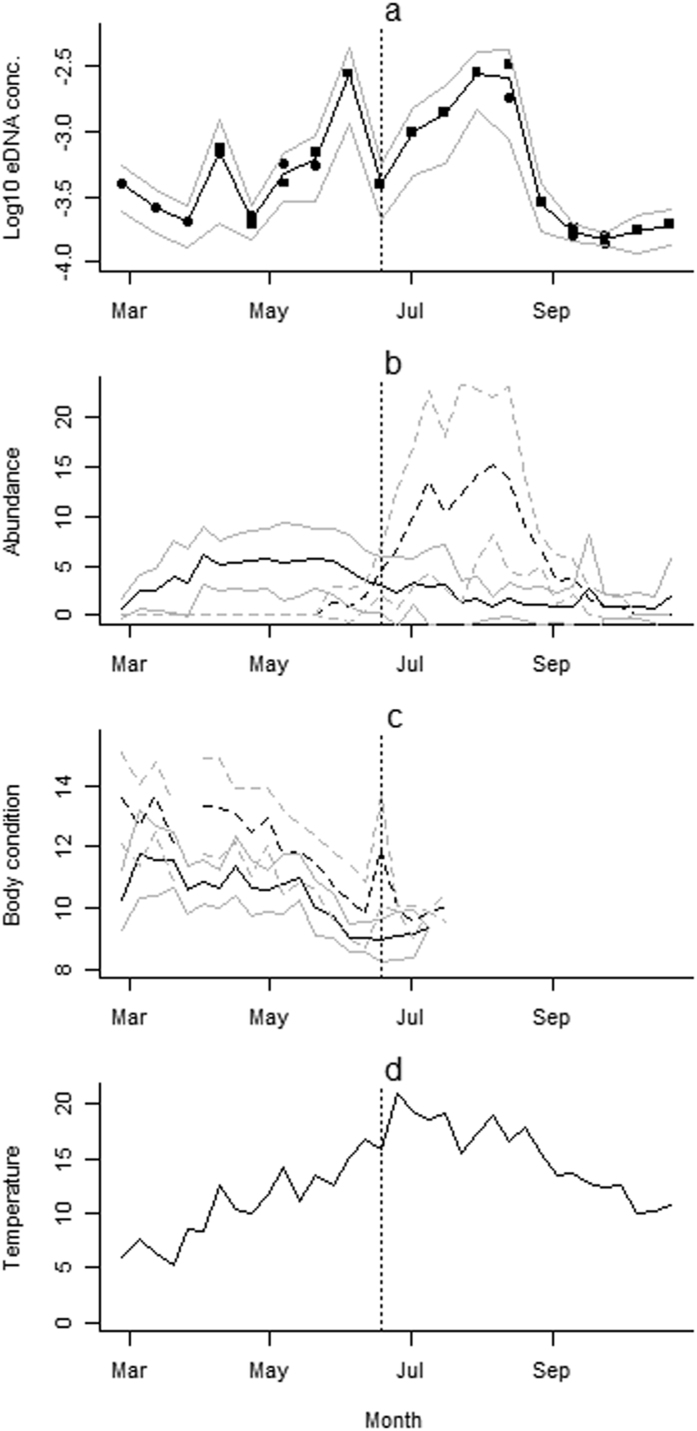
Seasonal variations in eDNA concentration, in relation to adult and larval population size, adult body condition and temperature. (**a**) Shows Log10 (x + 0.0001) of the mean eDNA concentration (ngμL^−1^), per pond (black line, solid circles collected using glass-microfiber filters, solid squares collected using precipitation in ethanol) with 95% confidence intervals (grey) across the eight ponds. (**b**) Shows the mean estimated population size per pond black (adults - solid line, larvae - broken line) with 95% confidence intervals (grey). (**c**) Shows mean body condition (males – solid line, females – dashed line) using the scaled mass index of adults caught each week throughout a survey season with 95% confidence intervals (grey). (**d**) Shows mean weekly temperatures in degrees Celsius through the study period. The vertical dotted line represents the end of the breeding season and the start of the post-breeding season, as related to the models described in Tables 1, 2.

Two distinct peaks were seen in eDNA concentration (Fig. 1). The first peak corresponded to the end of the adult breeding season in early June. The second peak was observed from mid-July to mid-August and corresponded with the peak in larval numbers. The influences on eDNA concentration over the breeding season (26 February to 18 June) were identified using the first set of models. The change in body condition measured by the Scaled Mass Index (SMI)^34^ fell from a peak on 6 March through the breeding season and continued to fall into the post-breeding season, with most of the decline occurring from 9 April through to 4 June. Both sexes showed declines in SMI score with females showing a slightly greater decrease than males (Fig. 1). The sharpest decline in body condition for both males and females occurred in the key breeding months of April and May. During the same core period of April and May the mean eDNA concentration rose considerably but adult population changed very little, and larvae were first identified in the ponds at the beginning of June. As would be expected, temperature and UV both increased as the breeding season progressed, from early spring into early summer. This resulted in the model with the greatest AIC support (∆AIC to second model = 0.5) comprising adult abundance, larval abundance, temperature, and male and female body condition as predictors of eDNA concentration (Table 1). Three other models were shown to have strong support (∆AIC ≤ 2) also detailed in Table 1.

Further analysis was undertaken on AIC importance weights for individual predictors over the breeding season, with female body condition (cumulative AIC weight = 0.99), larval abundance (cumulative AIC weight = 0.797) and air temperature (cumulative AIC weight = 0.79) strongly supported by the analysis, while male body condition (cumulative AIC weight = 0.428) and adult abundance (cumulative AIC weight = 0.44) were only somewhat supported by the analysis.

Influences on eDNA concentration after adult newts had finished breeding were examined through the second set of models, which included potential predictors from 18 June to 22 October. eDNA concentration increased dramatically between 18 June and 30 July, corresponding with an increase in mean larval abundance. During the same period adult abundance nearly halved, indicating that the increase in eDNA was more likely due to larval than adult influences. Temperature also increased through this period from a mean weekly temperature of 15.9 °C to over 19 °C for all of July. eDNA concentration remained high until the middle of August when it fell by over 90% between 13 August and 27 August, and continued to fall into the autumn. Metamorphosis of larvae from the ponds resulted in larval abundance falling over the same period. Temperature remained above 15 °C through August but then fell to below 10 °C in October. The model with the greatest AIC support (∆AIC = 4.82) included larval abundance and air temperature (Table 2) as predictors of eDNA concentration. No other models were shown to have strong support (∆AIC ≤ 2), but one was shown to have limited support (∆AIC ≤ 7) also detailed in Table 2.

Further analysis was undertaken on AIC importance weights for individual predictors for the post-breeding season, with larval abundance (cumulative AIC weight = 0.998), and temperature (cumulative AIC weight = 1.0) strongly supported by the analysis; no other variables were found to be strongly supported by the analysis. Sample collection method was not found to be a significant predictor of eDNA in any of the models.

## Discussion

Both laboratory and field studies have shown that an increase in abundance or density of target species can lead to an increase in either eDNA concentration^6^,^12^,^13^,^17^,^23^,^35^ or eDNA detectability^36^. Our results take this further by showing that the eDNA contribution from different life stages of a semi-aquatic species varies seasonally. Although it was artificially created, our replicated pond system was ideal for this work, as it allowed for truly replicated samples to be taken, with robust population estimates of naturally colonising newts obtained. eDNA concentration within the breeding season increases as females lose body condition through reproductive behaviour and laying eggs. Male body condition and adult abundance also have some influence on eDNA concentration during the breeding season but not to the same extent as other variables. After adult breeding activity has finished, eDNA increases again as larval abundance increases, but with temperature also having an influence at this time.

The amount of eDNA in the environment depends on both DNA release from organisms and eDNA degradation rate^22^. These rates are likely to vary seasonally in response to environmental changes and the ecology of the species^17^,^25^,^37^. Strong temporal increases in eDNA during months associated with breeding have been observed in the Eastern hellbender (*Cryptobranchus alleganiensis alleganiensis*)^19^ and Chinese and Japanese giant salamanders (*Andrias davidianus* and *A. japonicus* respectively)^20^. Doi *et al*.^16^ found that seasonal variations in eDNA concentration were related to total biomass, rather than abundance or behaviour, in stream dwelling fish^16^. Our data support this with an increase in eDNA concentration associated with both peak breeding and peak larval abundance.

Current eDNA survey protocols for great crested newts focus on the period adults are present in ponds^33^. In the past, positive great crested newt eDNA samples have been identified outside the breeding season^38^. We find a second period with high eDNA concentration at a time of year when adults are moving out of ponds into their terrestrial phase^39^. This post-breeding season spike can be attributed to other life stages, predominantly larvae, and the late August fall in eDNA, corresponds to the period larvae are metamorphosing and leaving the ponds^39^. Seasonal changes in eDNA therefore have implications for survey strategy. If the eDNA surveys are focused on assessing breeding rates, it may be more appropriate to attempt to target larvae by sampling over the post-breeding months. On the other hand, if surveys are aimed at determining occupancy by adults, this approach may be inappropriate. As with many other amphibians, great crested newts live in a metapopulations, where some ponds hold reservoirs of adults that are not breeding each year^40^. We have shown that one of the key influences on eDNA concentration after adults have finished breeding is larval abundance. Samples taken outside the core adult aquatic period may be useful in identifying successful breeding, due to the presence of larvae. However, in the cases of occupied but non-breeding ponds, samples in this period would likely return negative results, potentially missing important non-breeding sites for the species.

The relationship between eDNA water concentration and population size varies by season. For example, an increase in temperature is likely to influence both eDNA release, through higher activity levels^6^, and breakdown rates, with an increase in DNA degradation^25^,^28^. We found that temperature had a significant influence on eDNA concentration during both breeding and non-breeding periods. During the breeding season, temperature increased as did eDNA concentration, while during the post-breeding season (late summer and autumn) both eDNA and temperature decreased. This suggests that the seasonal activity of newts outweighs any influence temperature has on DNA degradation. High levels of rainfall would potentially dilute ponds thereby reducing eDNA concentration. However, we found that rainfall had no influence on eDNA concentration in our system. Although UV has been found to influence DNA^41^, its impact on degradation rates appears to be variable^3^,^28^,^42^,^43^,^44^,^45^. In the present study the correlation of UV with other potential environmental predictors means that separating its precise effects is confounded.

During the breeding season newts expend energy in courtship and reproduction, releasing pheromones^46^,^47^, spermatophores and eggs into the environment, all potentially directly or indirectly releasing DNA with them. The release of these products into the environment will not only lead to an increase in eDNA but it will reduce the mass of an individual and lead to a reduction in body condition. We observed a fall in both male and female body condition through the breeding season both of which were shown to be a significant influence on eDNA concentration. Reductions in male body condition were not as pronounced as for females and are likely to come from the release of spermatophores and expenditure of energy during courtship. The greater decline in female body condition and influence on eDNA over that from males is likely to be related to the greater loss of body mass due to egg production and laying.

Great crested newt females lay between 200 and 400 eggs per year^48^, which take between 15 and 20 days to develop^49^. However, this species suffers from a development arrest syndrome, with a chromosomal abnormality causing 50% of eggs to abort during the first two weeks of development^50^. As a result, this mortality is likely to release a large amount of eDNA into the water as eggs decompose. As egg production, egg abortion and hatching would be difficult to measure without destructive sampling, we believe that female body condition was a proxy measure for egg laying.

Can eDNA concentration be used as an index of relative abundance of target organisms rather than just presence or absence ? Our analyses – which provide more accurate estimates of adult and larval numbers than widely used visual count or trap-based survey methods – demonstrate that factors other than newt abundance influence the amount of eDNA present seasonally. Using eDNA to map population trends would therefore be problematical, although a relative abundance estimate between similar ponds, sampled concurrently under the same environmental conditions may be possible. Current traditional count-based population assessments from visual or trapping surveys for amphibians or other aquatic organisms suffer from the same issues, as detection rates may have poorly understood relationships to total population sizes and vary according to environmental conditions^51^. For stream fish, predictive models incorporating eDNA concentration are developing to identify detection probabilities, abundance, as well as eDNA production and discharge^52^. To apply this to population assessments of lentic, semi-aquatic amphibians, models would need to include seasonally variable DNA release and degradation rates, as well as taking into account multiple life stages. As these relationships become clearer, the role of eDNA in assessing populations is likely to become an increasingly valuable and cost-effective tool in assessing and mitigating the challenging problem of global amphibian declines.

## Methods

### Study Site

The study site was located at the University of Kent campus in Canterbury, UK. The site consists of eight identical ponds measuring 1 m × 2 m × 0.7 m deep constructed using PVC liner and a water volume when full of 600 L. The eight ponds are arranged in a grid pattern with approximately 3 m between each pond. All eight ponds can be considered to experience the same environmental conditions. All eight ponds had been in place for a minimum of six years at the time of the study and were allowed to be colonised naturally by the three species newts in the area^53^. All species could freely move from one pond to another and to immigrate or emigrate. Over the winter prior to the study, all eight ponds were drained, liners replaced and filled with tap water so that all ponds were identical at the start of the study.

### eDNA sampling

eDNA samples were collected from the eight ponds every 14 days from 26 February through to 22 October 2015. To avoid contamination, on each occasion eDNA samples were collected prior to the population monitoring. Two eDNA collection methods were used: (1) filtration of 1 L of sample water using a 0.7 μm glass-microfiber syringe filter (Sterlitech Corporation^®^, Kent Washington State, USA); and (2) precipitation of DNA from a 0.09 L sample volume in an ethanol, sodium acetate solution^54^. All field equipment was sterilised using 10% bleach, UV-Crosslinker or autoclave and sealed prior to transport to the study site, and a separate set of nitrile disposable gloves were used for each sample. Due to the small dimensions of each pond, a single 1 L surface sample, collected using a polypropylene wide mouth bottle, was deemed sufficient to provide a representative sample from each pond. The bottle was rinsed with pond water and used to stir the pond as suggested by Biggs *et al*.^33^ prior to being filled.

Filtered samples were collected using a 100 mL syringe. The sample was removed from the collection bottle, and then drawn through a 0.7 μm glass microfiber syringe filter. The process was repeated with the sample homogenised before filling each syringe. The process was repeated until 1 L had been filtered or two filter units had become blocked. Residual water was removed from the filter unit by passing two syringes of air through each unit. Both filter units were then sealed in bags prior to transport to the laboratory where they were stored at −20 °C until extraction.

Samples collected using precipitation in ethanol consisted of six, sterile 50 mL centrifuge tubes containing 33 mL of absolute ethanol and 1.5 mL of 3 M sodium acetate solution. All six tubes were filled from the collection bottle to make the volume in each up to 50 mL, using a sterile disposable plastic pipette. This equates to a total volume per sample of approximately 90 mL. Each sample was placed in a sealable bag for transport to the laboratory, where they were stored at −20 °C until extraction.

### Population assessments

The population in each pond was assessed using aquatic funnel traps^55^. Trapping commenced in the last week of February 2015 and continued weekly until the end of October 2015, encompassing the period adult and larval great crested newts are active^56^. Traps were left in place for between 11 and 12 hours overnight depending on the season. Ventral patterns of all adults caught were photographed and used for individual identification to allow for Capture-Mark-Recapture analysis to provide weekly detection probabilities^57^. Each adult was weighed on each capture event to the nearest 0.1 g, and snout-vent and tail length measured to the nearest 1 mm to assess body condition. To avoid contamination between ponds, surveyors wore disposable nitrile gloves that were changed between ponds. Additionally all bottle trapping equipment was sterilised at the start of the season with 10% bleach and dedicated equipment was used for sampling each of the eight ponds.

Torchlight counts of larvae were also conducted from the beginning of July onwards. This allowed calibration of the counts of larvae captured in the bottle traps at the same time. Torchlight counts involve shining a 1 million candle power torch through the surface of the water after dark. The light was moved systematically from one end of the pond to the other, counting all of individuals that could be seen within the water column. Due to the size of each of the study ponds and absence of vegetation, counts could be undertaken across the entire surface area and water column of each of the ponds.

### Laboratory protocol

DNA extractions were conducted in a UV sterilisable work station in a laboratory with dedicated equipment. All extractions were based on the DNeasy Blood & Tissue Extraction kit (Qiagen^®^, Hilden, Germany) with amended protocols as outlined in the Supplementary Material. Periodic extraction blanks for both methods were undertake through the laboratory phase of the project to check for equipment contamination, and were all negative.

Real-Time qPCR was performed on all samples in a separate lab from DNA extraction and in a dedicated UV-sterilisable work station. qPCR was performed using previously published primers and hydrolysis probe^12^ and qPCR assay and cycle condition^54^ using a CFX Connect Real-Time PCR detection system (BIO-RAD^®^ Hercules, California, USA). Eight qPCR replicates were performed per sample. qPCR standards were created from a serial dilution of a great crested newt tissue extract, quantified using a Qubit^®^ 2.0 flurometer (Life Technologies^TM^, Carlsbad, California, USA) with Double Stranded DNA High Sensitivity Kit following manufacturers’ instructions, qPCR negative controls were also included in each run. The median value for the eight qPCR replicates was taken forward into the analysis for each sample. eDNA was found in all ponds, but not in each calendar week, with concentration varying between zero and 0.00845 ngμL^−1^. The mean R-squared value of all qPCR standard curves was 0.99 and the efficiency was 90.3%.

The limit of detection (LOD) and limit of quantification (LOQ) were calculated through qPCR from a serial dilution of a tissue extract from a great crested newt. The LOD related to the minimum concentration amplification was observed, while the LOQ was assigned to the minimum level that exhibited a high degree of conformity between qPCR replicates^24^. The LOD was found to be less than 10^−7^ ngμL^−1^, with an LOQ of 10^−5^ ngμL^−1^.

Great crested newt eDNA was detected in some or all ponds on each survey occasion. Eleven out of 200 eDNA samples analysed returned as negative. Negative results were split between both survey methods and were only found when eDNA concentrations were low either towards the start or end of the study.

### Environmental Data

Mean temperature as well as UV levels for the 14 days between sampling were generated for the study site as a whole. Air temperature was recorded from the site hourly using a Tinitag^®^ Plus 2 – TGP-4017 (Gemini Data Loggers, Chichester, UK) commencing on the 30 January. UV was recorded on a TR-74Ui – Illuminance UV Recorder (T&D Corporation^®^, Nagano, Japan) at hourly intervals, from 17 February. An indication of the level of rainfall that occurred between each survey period was collected using a standard rain gauge, emptied at the time of the visual surveys.

### Analysis

Losses of body mass during the breeding season are associated with egg deposition (females), spermatophore production (males) and utilization of fat reserves for breeding activity. Body condition estimates were generated using the Scaled Mass Index (SMI)^34^. The mean of the SMI values for all individuals caught each week were taken to produce each weekly value. SMI values could only be generated until the middle of July due to low adult numbers caught beyond that point. This was done for males and females separately as well as both sexes combined.

The Cormack-Jolly-Seber model and Program MARK^57^ were used to generate a detection probability each week for adults captured in traps. The best fitting model was phi(.)p(t), or constant survival with variable detection probability. Detectability varied each week and ranged from 0 to 1 with the majority of results falling between 0.3 and 0.6, with outliers from this range only found in weeks when few individuals were caught. A single detection probability was generated for the larvae, using torchlight counts, as using capture mark recapture was not a viable option for larvae. Using ponds with high visibility, which allowed the entire pond to be observed, the number of larvae captured in traps was divided by the number of larvae counted in the torchlight surveys. This approach is appropriate in the case of this study due to the small size of the ponds allowing the entire pond to be searched by torchlight. A fixed detection probability of 0.39 was used in all weeks for two reasons. Firstly, the low number of individuals in the last few weeks of the study skewed detectability estimates. Secondly, torchlight counts only started on 9 June, after the first larvae were caught in traps, therefore no detection probability could be generated for the weeks before the introduction of torchlight counts. The population size for each pond in each week was estimated by multiplying the number of newts caught in traps by the reciprocal of the detection probability^21^. Population estimates and body condition scores are only included in the analysis for the weeks eDNA was collected.

### Statistical analysis

eDNA concentrations were transformed prior to analysis using y = log10 (x + 0.0001) to ensure normality. All statistics were conducted using linear mixed effect models (LMM)^58^ using R version 3.1.3^59^ and package nlme^60^, LMM were chosen to account for the repeated measures on the same ponds through the season (treated as a random effect). Akaike’s Information Criterion (AIC) was used to assess support for different models using package MuMIn^61^. Models with a ∆AIC of ≤2 were considered to have substantial support, while models with a ∆AIC of ≤7 were considered to have some support^62^. Using the full set of models, Akaike importance weights for predictors were calculated as measures of parameter importance, by summing the Akaike weights for each model containing that variable^62^,^63^. Parameters were classed as strongly supported by our models if they were significant in all strongly supported models (∆AIC of ≤2) and had a cumulative Akaike weight of >0.75^62^. Parameters were considered somewhat supported if they were significant in any of the strongly supported models (∆AIC of ≤2) regardless of Akaike weight^62^.

Two models were run, because different factors potentially influence eDNA concentration at different times of year: (1) a model encompassing the core adult aquatic period (26 February to 18 June); and (2) a model encompassing the post-breeding season when most adults will be on land (18 June to 22 October). A single model would be inappropriate because estimates for body condition were only available for those weeks when adults were in the ponds in high numbers, and would lead to a high degree of non-random missing data biasing the output. The first set of models therefore incorporated the breeding season (i.e. 26 February to 18 June), and comprised nine eDNA sampling occasions across 18 weeks. These models were constructed with “Pond” as the random variable to account for repeated sampling, and combinations of adult abundance, larval abundance, male body condition, female body condition, combined body condition, calendar week, collection method, air temperature, water temperature, rainfall and UV included as covariates. All variables were treated as continuous covariates with the exception of collection method which was nominal. Correlation coefficients were examined for covariates included in all strongly supported models (∆AIC of ≤2), a pair of covariates were considered to be highly correlated with a correlation coefficient of >0.7^64^. This was found to be the case for UV and female body condition (r = 0.868 in the top model), and as a result UV was excluded from the analysis. No other pairs of covariates were found to be above this threshold.

The second set of models explored variation in eDNA concentration outside the breeding season (i.e. 18 June to the 22 October), including ten eDNA sampling occasions across 19 weeks, with “Pond” again used as the random variable to account for repeated sampling. Adult abundance, larval abundance, eDNA collection method, air temperature, rainfall, UV, and calendar week, were all included as covariates. All variables were treated as continuous covariates with the exception of collection method which was nominal. Correlation coefficients were examined for covariates included in all strongly supported models (∆AIC of ≤2), a pair of covariates were considered to be highly correlated with a correlation coefficient of >0.7^64^.This was found to be the case for UV and calendar week (r = 0.960 in the top model), and as a result UV was excluded from the analysis, no other pairs of covariates were found to be above this threshold.

Collection method (i.e. ethanol precipitation versus glass-microfiber syringe filtration) was included as a variable in all of the models to check that there was no method-related bias. This was subsequently corroborated, with paired sample analysis showing no difference in eDNA extract concentration between the two methods (Buxton *et al*. in prep.).

### Ethics Statement

The experimental procedure was approved by the School of Anthropology and Conservation (University of Kent) Research and Ethics Committee, with disturbance and handling of live animals undertaken under EPS Licence 2014-5025-CLS-CLS issued by Natural England, in accordance with the conditions of the licence.

### Data accessibility statement

This statement confirms that, should the manuscript be accepted, then data supporting the results will be archived in the Kent Academic Repository.

## Additional Information

**How to cite this article**: Buxton, A. S. *et al*. Seasonal variation in environmental DNA in relation to population size and environmental factors. *Sci. Rep.* **7**, 46294; doi: 10.1038/srep46294 (2017).

**Publisher's note:** Springer Nature remains neutral with regard to jurisdictional claims in published maps and institutional affiliations.

## Supplementary Material

Supplementary Methods

## Figures and Tables

**Table 1:** Linear mixed effect models showing influences on eDNA concentration in the breeding season (26 February to 18 June).

Predictor	Random	Value	SE	DF	t-value	p-value	AIC	AICc	∆AIC	Weights
Adult Abundance	Pond	0.029	0.008	83	3.68	0.0004	97.91	99.6	0.00	0.263
Larval Abundance		0.043	0.021	83	2.01	0.0481				
Male Body Condition		−0.189	0.072	83	−2.61	0.0108				
Female Body Condition		−0.328	0.065	83	−5.02	<0.0001				
Temperature		−0.117	0.022	83	−5.32	<0.0001				
Larval Abundance	Pond	0.054	0.020	85	2.65	0.0095	99.13	100.1	0.50	0.204
Female Body Condition		−0.398	0.063	85	−6.31	<0.0001				
Temperature		−0.101	0.023	85	−4.33	<0.0001				
Adult Abundance	Pond	0.026	0.008	84	−4.99	<0.0001	99.13	100.4	0.83	0.174
Larval Abundance		0.066	0.020	84	3.32	0.0013				
Female Body Condition		−0.404	0.061	84	−6.66	<0.0001				
Temperature		−0.113	0.0223	84	3.223	0.0018				
Female Body Condition	Pond	−0.176	0.039	87	4.53	<0.0001	100.79	101.2	1.66	0.115

All models showing substantial support based on ∆AIC shown.

**Table 2:** Linear mixed effect models showing influences on eDNA concentration post-breeding season (18 June to 22 October).

Predictor	Random	Value	SE	DF	t-value	p-value	AIC	AICc	∆AIC	Weights
Larval Abundance	Pond	0.013	0.002	94	5.36	<0.0001	100.74	101.4	0.00	0.898
Temperature		0.056	0.014	94	6.13	<0.0001				
Collection Method	Pond	0.065	0.079	93	0.82	0.4166	105.31	106.2	4.82	0.081
Larval Abundance		0.012	0.002	93	5.29	<0.0001				
Temperature		0.088	0.014	93	6.16	<0.0001				

All models showing substantial or some support based on ∆AIC shown.
