# Supplementary material for: Seasonal variation in environmental DNA in relation to population size and environmental factors
Source: Sci Rep. 2017 Apr 10;7:46294. doi: 10.1038/srep46294 (PMC5385492; doi:10.1038/srep46294)
Supplement: Supplementary Methods [file srep46294-s1.pdf]

Seasonal variation in environmental DNA in relation to population size and environmental factors

Andrew S. Buxton*, Jim J. Groombridge, Nurulhuda B. Zakaria, Richard A. Griffiths.

All Authors - Durrell Institute of Conservation and Ecology, School of Anthropology and Conservation, University of Kent, Marlowe Building, Canterbury, Kent, United Kingdom, CT2 7NR, UK

Authors email - Andrew S. Buxton (asb40@kent.ac.uk); Jim J. Groombridge (J.Groombridge@kent.ac.uk); Nurulhuda B. Zakaria (nbz3@kent.ac.uk); Richard A. Griffiths (R.A.Griffiths@kent.ac.uk).

Supplementary Material

Extraction protocols

The glass-microfiber filters were extracted in a fume hood, sterilised with a 10% bleach solution and UV-light the filter paper was removed from the sealed syringe filter holder using sterilised wire cutters and sterilised forceps. Once removed the filters were cut into strips approximately 3mm in width with each filter placed length ways into a separate 1.5 mL microcentrifuge tube. As a result for the digestion step each sample consisted of two microcentrifuge tubes, one for each of the two filters. 675 µL of the ATL buffer from the DNeasy Blood & Tissue kit was added to each tube; it was then vortexed for 15 seconds to mix before 20 µL of Pro K was added and again vortexed. The samples were then incubated on a rotating block, for 3 hours at 56°C or overnight at 37°C. Following incubation the liquid was then transferred to a fresh microcentrifuge tube; the two digestion reactions for a sample were combined at this stage. 200 µL of AL buffer and 200 µL of ice cold absolute ethanol was added to each tube and vortexed for 15 seconds to facilitate DNA precipitation. 650 µL of the extraction solution was transferred into a DNeasy Blood and Tissue kit Mini spin column and

centrifuged at 8000rpm for one minute with the flow through discarded. This was then repeated using the same mini spin column until the entire sample had been passed through. DNA extraction continued as per the DNeasy Blood and Tissue kit manufacturers' protocol, eluting into 200 µL of the elution buffer.

Extraction from ethanol precipitation samples was undertaken using a modified protocol from Biggs (*et al.*)[1]. The mixture was centrifuged at 10,020g, (8500rpm) for 35 minutes and the supernatant discarded. The remainder of the extraction protocol was conducted as per Biggs (et al.)[1].

References

1. Biggs, J. *et al.* Using eDNA to develop a national citizen science-based monitoring programme for the great crested newt (Triturus cristatus). *Biol. Conserv.* **183,** 19–28 (2015).
